# Re-Evaluation of PD-1 Expression by T Cells as a Marker for Immune Exhaustion during SIV Infection

**DOI:** 10.1371/journal.pone.0060186

**Published:** 2013-03-28

**Authors:** Jung Joo Hong, Praveen K. Amancha, Kenneth Rogers, Aftab A. Ansari, Francois Villinger

**Affiliations:** 1 Department of Pathology and Laboratory Medicine, Emory University School of Medicine, Atlanta, Georgia, United States of America; 2 Division of Pathology, Yerkes National Primate Research Center, Atlanta, Georgia, United States of America; St. Jude Children's Research Hospital, United States of America

## Abstract

PD-1 expression is generally associated with exhaustion of T cells during chronic viral infections based on the finding that PD-1 expressing cells respond poorly to antigen activation and blockade of PD-1/PD-ligand interaction restores such antigen specific responses in vitro. We tested this hypothesis by examining PD-1 expression on virus-specific CD8 T cells and total T cells in vivo to determine whether PD-1 expression constitutes a reliable marker of immune exhaustion during SIV infection. The expression of PD-1 and Ki67 was monitored longitudinally on T cell subsets in peripheral blood, bone marrow, lymph node and rectal biopsy specimens from rhesus macaques prior to and post infection with pathogenic SIVmac239. During the course of infection, a progressive negative correlation was noted between PD-1 density and Ki67 expression in p11CM^+^ CD8^+^ T cells, as seen in other studies. However, for total and memory CD4 and CD8 T cells, a positive correlation was observed between PD-1 and Ki67 expression. Thus, while the levels of non-proliferating PD-1^+^ p11CM^+^ CD8 T cells were markedly elevated with progressing infection, such an increase was not seen on total T cells. In addition, total memory PD1^+^ T cells exhibited higher levels of CCR5 than PD-1^−^ T cells. Interestingly, few PD-1^+^ CD8^+^ T cells expressed CCR7 compared to PD-1^+^ CD4 T cells and PD-1^−^ T cells. In conclusion, overall PD1^+^ T cells likely represent a particular differentiation stage or trafficking ability rather than exhaustion and in the context of chronic SIV infection, the level of PD-1 expression by T cells does not by itself serve as a reliable marker for immune exhaustion.

## Introduction

Programmed cell death 1 (PD-1) is a member of the CD28 family, which modulates T cell function [1] and is primarily up-regulated on the surface of CD4 and CD8 T cells upon activation [2]. PD-1 interacts with its ligands PD-L1 or PD-L2 and this engagement induces tyrosine phosphorylation of the cytoplasmic domain of PD-1. This process recruits tyrosine phosphatases which dephosphorylate TCR proximal kinases to limit the TCR/CD28 signal transduction. In this context, PD-1 cross linking results in impairment of T cell-mediated immune responses to tumors and chronic viral infections. Blocking of the PD-1/PD-L1 pathway in LCMV infected mice with the use of anti-PD-L1 monoclonal antibody was shown to restore function in exhausted CD8^+^ T cells which led to a significant reduction of viral load [3]. Similar findings have been observed in other chronic viral infections, such as human T cell lymphotrophic virus (HTLV), hepatitis C virus (HCV), and human immunodeficiency virus (HIV)/simian immunodeficiency virus (SIV) [3]–[6] and more recently in patients with various forms of advanced cancers [7], [8]. These findings indicate that the expression of PD-1 by T cells distinguishes physiologically “activated” cells from “exhausted” T cells as a result of persistent antigenic stimulation.

Although PD-1 expression by antigen specific CD8 T cells has been associated with an exhausted phenotype, the phenotypic and functional characteristics of PD-1 expressing conventional CD4 and CD8 T cells under normal physiological conditions and chronic antigen persistence remain to be addressed. Furthermore, several lines of experimental evidence argue that PD-1 expression alone should not be regarded as a definitive marker for exhausted cells. First, PD-1 is an activation marker of CD4 and CD8 T cells and similar to CTLA-4, may be upregulated early to potentially prime a negative regulatory feedback mechanisms to limit inflammation. PD-1 is induced by antigen specific and non-specific stimulation on T cells [2], [9], [10], yet additional conditions may be needed to fully engage the regulatory pathway or alternatively, ligation does not fully occur due to cell traffic. Second, recent work has shown that, in healthy individuals, PD-1^+^ cells do not exhibit gene expression profiles characteristic of exhausted T cells nor are they functionally impaired [11]. Third, the expression of activation markers, such as CD38 and HLA-DR, positively correlate with PD-1 expression on CD8 T cells in HIV-1 infected patients [12], [13]. Nevertheless, the level of PD-1 on CD4 and CD8 T cells has still been equated to immune exhaustion in recent HIV clinical studies [14]–[16]. To further address this issue, particularly in the context of chronic lentiviral infection, we investigated the proliferative status (Ki67 index) of the global population of PD-1 expressing CD4 and CD8 T cells during chronic SIV infection, as a model for HIV infection.

## Materials and Methods

### Animals

Twenty adult Indian rhesus macaques were used as described previously [17]. The monkeys were typed for a select number of major histocompatibility complex antigens utilizing PCR techniques with allele-specific primers specific for Mamu-A*001, -B*001, B*008 and B*017, as previously described [18]. Monkeys expressing the B*008 and B*017 MHC alleles were excluded from this study since these alleles are associated with better viral control [19]. The selected monkeys included 7 Mamu-A*001 and 13 non-Mamu-A*001 that were placed into 2 groups with group I receiving anti-retroviral drugs (with 4 Mamu-A*001^+^ monkeys) and the other serving as a control (3 Mamu-A*001^+^ monkeys). All 20 animals were inoculated with 200 TCID_50_ (50% tissue culture infective dose) of SIVmac239 intravenously. Blood, lymph nodes and colorectal biopsies were obtained from each of these 20 animals at various times pre and post infection (pi). Group I (n = 10) were administrated 9-(2-phosphonyl-methoxyprophly) adenine (PMPA; 30 mg/kg) and Racivir (15 mg/kg) subcutaneously daily for 28 days starting on 112 day pi after reaching viral load set-point. Group II (n = 10) served as controls.

### Ethics statement

All animals were born and maintained at the Yerkes National Primate Research Center of Emory University in accordance with the regulations of the Committee on the Care and Use of Laboratory Animal Resources. The animals are fed monkey diet (Purina) supplemented daily with fresh fruit. Additional enrichment is provided and overseen by the Yerkes enrichment staff and animal health is monitored daily by the animal care staff and veterinary personnel. Animals that reached IACUC defined endpoints, including pain or stress that could not be alleviated therapeutically were humanely euthanized with an overdose of barbiturate consistent with the recommendation of the American Veterinary Medical Association. The Yerkes National Primate Research Center is fully accredited by the Association for Assessment and Accreditation of Laboratory Animal Care International. All experiments were reviewed and approved by the Emory institutional animal use and care as well as biosafety review Committees.

### Quantitation of SIV RNA in Plasma

Plasma SIV viral load was determined by quantitative RT-PCR by the Emory, NIH CFAR sponsored Virology Core Laboratory [20].

### Preparation of mononuclear cells from blood, peripheral lymph nodes and colorectal biopsies

Peripheral blood mononuclear cells (PBMC) were isolated from fresh blood using standard Ficol-Hypaque gradient. A mononuclear cell suspension was prepared from inguinal LN and colorectal biopsy samples as previously described [21]. Lymph node cells were collected by mincing the tissues and passing the cells through a 40 µm cell strainer (Fisher Scientific). Colorectal biopsies were treated with collagenase and DNase at room temperature for 2 hours and separated by passage through a 10-cc syringe fitted with decreasing needle bores. Cells were purified using discontinuous percoll gradients (60∶30%). All lymphocytes were >90% viable by trypan blue exclusion.

### Flow cytometry

Peripheral blood monocuclear cells (PBMC), lymph node and colorectal cells were stained with antibodies as described previously [17], [22]–[24]. In order to study the expression of chemokine receptors on T cells, whole blood prior to lysing was used [25]. Briefly, 150 µl blood aliquots and 1 million cells isolated from tissues were incubated with a predetermined optimal concentration of the following antibodies: anti-CD3-Alexa fluor 700 (clone SP34-2), anti-CD8-Pacific Blue (clone RPA-T8), anti-CCR5-APC (clone 3A9), anti-and CD95-phycoerythrin (PE)-Cy5 (clone DX2), all from BD Biosciences. Anti-CD4-AmCyan (clone L200) was obtained courtesy of the NIH Nonhuman Primate Reagent Resource. Anti-PD-1-PE-Cy7 (clone EH12.2H7) and the anti-CCR7-APC-Cy7 (clone 3D12) antibodies were purchased from Biolegend. Anti-NKG2A (clone Z199) and anti-CD28 (clone CD28.2) were obtained from Beckman-Coulter (Brea, CA). For the p11CM tetramer staining, cells were incubated with PE or APC-conjugated p11CM tetramer (courtesy, The NIH Tetramer Facility, Emory University) for 1 hour at 37°C. After lysing with BD FACS™ lysing solution, blood cells were washed with PBS containing 2% fetal bovine serum (FBS). PBMC and cells from tissues were washed without this lysing step. They were then permeabilized with BD cytofix/cytoperm™ for 20 minutes and washed with perm wash buffer. Finally, they were incubated with anti-Ki67-FITC and for purposes of control isotype-FITC, washed, and fixed with 1% paraformaldehyde (PFA). Data were acquired on a LSRII flow cytometer (BD Bioscience) and the data obtained analyzed using FlowJo software (version 9.2 Tree Star, Ashland, OR).

### Carboxyfluorescein diacetate N-succinimidyl ester (CFSE) assay

Labeling of PBMC with CFSE was performed as previously described [22]. In brief, 2 mls of a 40 µM solution of CFSE (Invitrogen, Carlsbad, CA) was added to 10^7^ cells that were suspended in 2 ml of PBS containing 0.1% BSA. The cells were incubated at 37°C for 10 minutes. After the incubation, 10 ml of ice-cold RPMI-1640 medium containing 10% FBS, 100 units/ml penicillin/streptomycin and 2 mM L-Glutamine (complete medium) were added and the cells were washed twice in complete medium. The cells were then transferred to a 24-well plate (1 ml/well), with 1 mg/ml of anti-CD28a and anti-CD49d each as co-stimulants, incubated at 37°C, 7% CO_2_ with or without SIV specific or control peptides (0.1 µg/ml). After 7 days, cells were stained first with the Live/Dead marker (Alexa 430 Invitrogen A10169) at room temperature for 30 min. Next the cells were incubated with alexa flour 700-conjugated anti-CD3 (clone SP34-2, BD), Peridinin-chlorophyll protein-conjugated anti-CD4 (clone L200, BD), BD Horizon V450-conjugated anti-CD8 (clone RPA-T8, BD) and phycoerythrin (PE) Cy7-conjugated anti-PD1 (clone EH12.2H7 eBiosciences) for 15 min at room temperature, centrifuged, washed and re-suspended in FACS buffer and analyzed. Data were acquired and analyzed as described above.

### Statistical methods

All statistical analyses were performed using GraphPad Prism (version 5.03) and GraphPad instat (version 3.10). For the comparison of data obtained at two time points, the Mann-Whitney ‘U” test (Two-tail *p* value) and the Wilcoxon matched pairs test (Two-tail *p* value) was used. The level of correlation was assessed by Spearman's rank correlation test. A p-value of less than 0.05 was considered statistically significant.

## Results

### Exhaustion of antigen-specific CD8 T cells throughout the chronic stage of SIV infection

**Figure 1:**
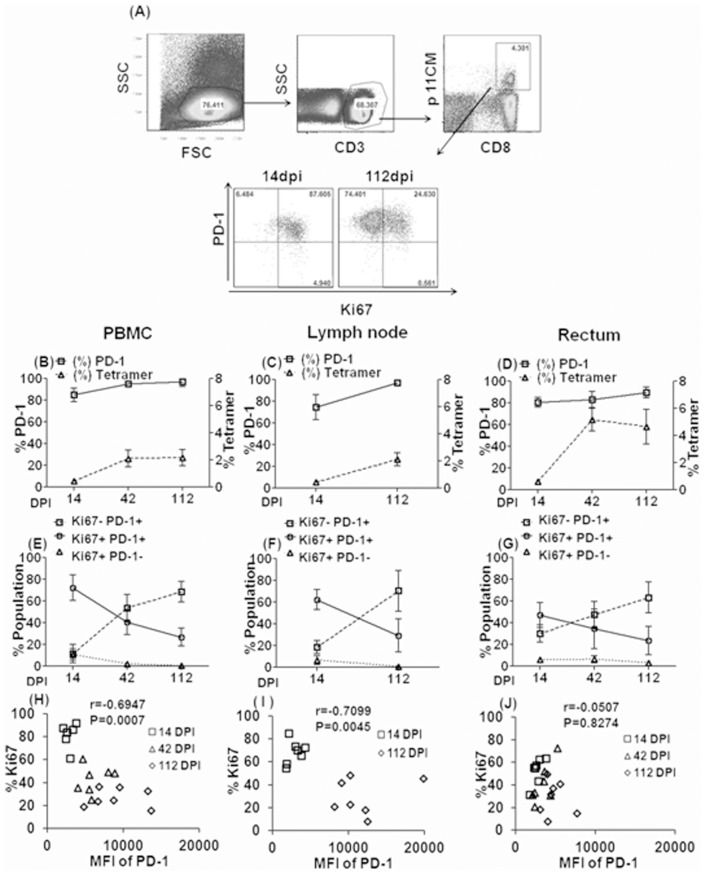
Longitudinal analysis of SIVgag-specific CD8 T cells in seven Mamu A*01 rhesus macaques infected with SIVmac239. The gating strategy and representative flow cytometry showing the percentage (Mean +/− S.D.) of PD-1 expressing p11CM^+^ CD8^+^ T cell (Ki67**^+^** vs Ki67^−^) in PBMC at 14 and 112 dpi is shown in (A). The frequency of p11CM^+^ CD8^+^T cells and their PD-1 expression in PBMC (B), lymph node (C) and colorectal tissues (D) at 14, 42, 112 dpi are shown. The frequency of Ki67^−^ PD-1**^+^**, Ki67^+^ PD-1**^+^** and Ki67^+^ PD-1^−^ cells in p11CM^+^ CD8^+^ T cell in PBMC (E), lymph node (F), and colorectal tissues (G) following SIVmac239 infection are shown. The relative density of PD-1 expression (MFI) by p11CM^+^ CD8^+^T cells is plotted as a function of proliferative capacity based on Ki67 expression in PBMC (H), lymph node (I), and colorectal tissues (J) at 14, 42, and 112 dpi. PBMC, lymph node, and colorectal cell samples from Seven 7 Mamu-A*001 animals were included in these analyses.

During chronic viral antigenic stimulation, PD-1 can be highly upregulated, which when cross-linked by its cognate ligands, leads to T cell exhaustion characterized by loss of the proliferative capacity and cytokine production in response to specific antigenic stimulation [26]. To analyze antigen-specific CD8 T cells in a non-human primate model, we studied seven Mamu-A*001 rhesus macaques among our twenty animals. The frequencies of p11CM^+^ CD8^+^ T cell in PBMC, lymph node and colorectal biopsy samples were below the level of detection before infection but clearly identifiable on the gated population (Fig. 1A) of CD3^+^/CD8^+^ cells during acute infection and were relatively stable during chronic infection (Figure 1B, C and D). The majority of these p11CM^+^ CD8 T cells were also positive for PD-1 (66.89% to 99.8%, Figure 1B, C and D). Next, we measured the expression of Ki67, a surrogate marker for T cell proliferation [27], [28] (23) on PD-1 expressing p11CM^+^ CD8 T cells during acute and chronic SIV infection. The frequencies of Ki67 expressing PD1^+^ p11CM^+^ CD8 T cells in PBMC, lymph node and the rectal biopsy samples was very high (72.3±11.9 %, 68.5 ±10.2 and 52.7 ± 11.6, respectively) during acute infection and then decreased over time post infection. The frequency of Ki67 negative p11CM^+^ CD8 T cells, on the other hand, dramatically increased during chronic infection (Figure 1E, F and G), suggesting indeed, increased immune exhaustion of these SIV specific effector cells in vivo. We also evaluated the relationship between the expression of this proliferation marker and PD-1 on these cells. As expected, PD-1 density of p11CM^+^ CD8 T cells negatively correlated with Ki67 expression over time post infection (from 14 to 112 dpi), except for the colorectal samples (Figure 1H, I and J) suggesting that PD-1 expressing virus-specific CD8 T cells become functionally impaired in their ability to proliferate with progressive infection.

### Proliferation status of global T cells and PD-1 expression during chronic SIV infection

**Figure 2:**
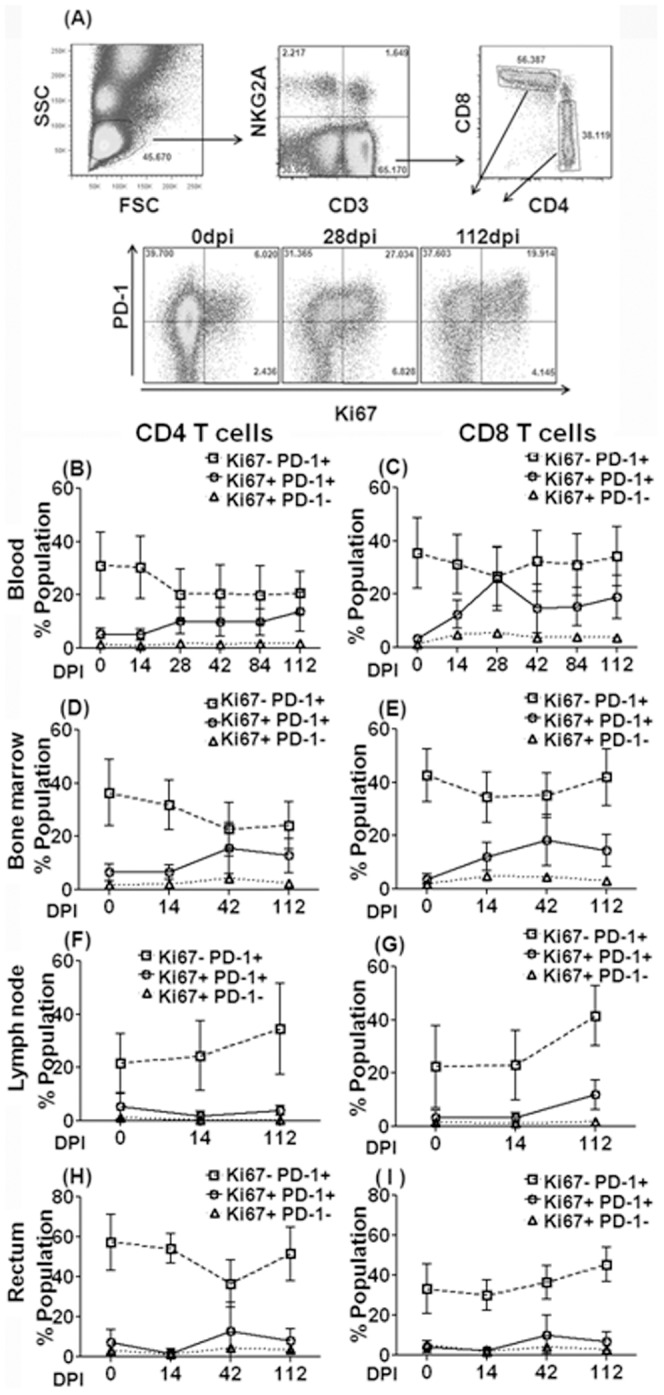
Longitudinal analysis of PD-1 and Ki67 expression on CD4^+^ and CD8^+^ T cells in blood and tissues of twenty SIVmac239-infected rhesus macaques. The gating strategy utilized and representative flow cytometry profiles showing the percentage (Mean +/- S.D.) of PD-1^+^ CD4 and CD8^+^ T cell (Ki67^+^ vs Ki67^−^) in whole blood at 0, 28, 112 dpi shown in (A). The frequency of PD-1^+^ Ki67^+^, PD-1^+^ Ki67^−^ and PD-1^−^ Ki67^+^ cells in CD4^+^ (B, D, F and H) and CD8^+^ T cells (C, E, G, and I) prior to and following SIVmac239 infection in whole blood (B and C), bone marrow (D and E), lymph node (F and G), and colorectal tissues (H and I). Whole blood, bone marrow, lymph node, and colorectal cell samples from twenty animals were used for the analyses, except for the lymph node at 0 dpi (n = 13).

We next examined the expression of PD-1 on the gated populations (Fig. 2A) of total CD3^+^ CD4^+^ and CD3^+^ CD8^+^ T cells (gating out all other cell lineages including monocytes and NKG2a^+^ cells), the gated populations of CD3^+^/NKG2a^+^ and the CD3^−^/CD8^+^/NKG2a^+^ (NK cells) from each of the 20 rhesus macaques prior to and post (day 0 to day 112 pi) SIV infection. All twenty rhesus macaques exhibited high plasma viral loads that peaked between 14 and 21 days post infection (4.3×10^5^ to 3.5×10^7^ viral RNA copies/ml plasma) with sustained plasma viral load set point >10^5^ viral copies, except for one animal which controlled viremia (Figure S1A). In agreement with our previous report [22] and others [29], PD-1 expression on total CD4 and CD8 T cells was not markedly altered during chronic SIV infection, compared with expression levels measured before infection. Over the chosen time points, wide variations were noted in the density of PD-1 expression on peripheral CD4^+^ T cells, CD8^+^ T cells and CD3^+^ NKG2a^+^ cells among the different animals, while comparatively little expression was noted on NK cells (Figure S1). Next, we analyzed the frequencies of Ki67 expression by total PD-1^+^ CD4^+^ (Fig. 2 B, D, F and H) and CD8^+^ (Fig. 2 C, E, G and I) T cells in peripheral blood, bone marrow, lymph node, and rectal biopsy samples. Most Ki67^+^ T cells were also PD-1^+^ in vivo, only a minor percentage of Ki67^+^ T cells were PD-1^−^ throughout the course of infection (Figure 2). Of interest was the observation of a marked increase in the frequency of Ki67^+^PD-1^+^ cells in blood and bone marrow but not the colorectal biopsy tissues in samples obtained from acute to the chronic phase of infection (Figure 2 B, C, D, E, H, and I), while in lymph nodes such increase was only noted for CD8^+^ T cells. In contrast to the other tissues, the frequencies of CD4 and CD8 T cells expressing PD-1 but not Ki67 increased in lymph nodes while these values decreased or remained stable in blood, bone marrow and colorectal samples suggesting tissue site specific differences in the frequencies of proliferating T cell subsets [17]. For NKG2A^+^ CD3^−^ NK cells, only Ki67^+^ PD-1^−^ cells, not PD-1^+^ cells dramatically increased at 14 dpi and remained elevated during chronic infection (Figure S2E to H). Similar findings were noted in tissues, although the magnitude of the changes was most marked in blood. CD3^+^ NKG2a^+^ cells showed an early and sustained increase in both Ki67^+^ PD-1^+^ and PD-1^−^ T cells in the blood while in tissues these changes were non-detectable.

**Figure 3:**
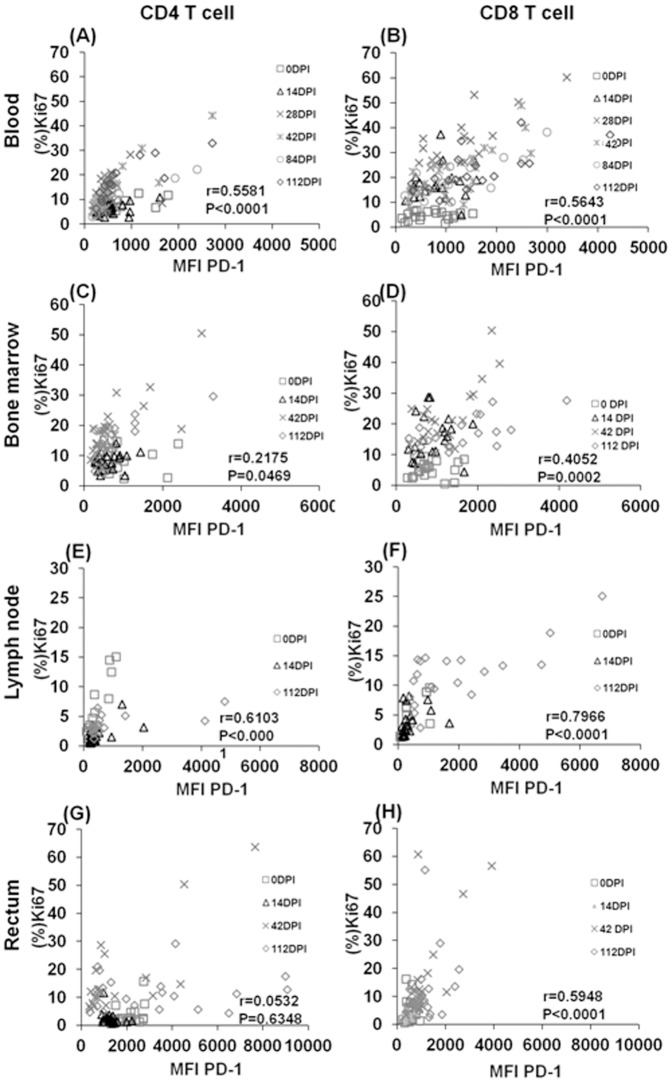
Longitudinal analysis of correlations between the density of PD-1 expression (MFI) by CD4 and CD8 T cells with the frequencies of Ki67 expressing cells in blood and tissues during SIV infection. PD1 expression positively correlated with Ki67 expression on CD4 (A, C, E and G) and CD8 T cells (B, D, F, and H) during the chronic infection, except for CD4 T cells from the colorectal tissues. Data of each individual animal pre- and post- SIV infection are shown. The correlation was assessed by Spearman's rank correlation test. A p-values of less than 0.05 were considered statistically significant. Whole blood, bone marrow, lymph node, and colorectal cell samples from twenty animals were used for the analyses, except for the lymph node at 0 dpi (n = 13).

Analyses of Ki67 and PD-1 expression on CD4 and CD8 subsets in peripheral blood, bone marrow, lymph node, and colorectal biopsy samples demonstrates that a significant positive correlation exists in their expression by both CD4 and CD8 T cells post SIV infection (Figure 3A to H) in blood and all tissues examined with the exception of Ki67 expression by the colorectal CD4^+^ T cells (Figure 3G). However, this latter data needs to be interpreted in light of the marked depletion of CD4^+^ T cells in gut post infection and the presence of detectable levels of PD-1^hi^ CD4^+^ cells in the lamina propria of this tissue, especially at the chronic stage of infection (ongoing investigations). Thus, following SIV infection, proliferative activity positively correlated with PD-1 expression on global populations of both CD4 and CD8 T cells, unlike antigen specific T cells such as p11CM^+^ CD8^+^ T cells, for which a negative correlation was evident.

To confirm that chronically proliferating T cells are indeed positive for PD-1, we used a carboxyfluorescein succinimidyl ester (CFSE) assay to examine the percentage of PD-1 expression on proliferating CD4 and CD8 T cells from seventeen RM chronically infected with SIV (231 dpi). Following in vitro re-stimulation with gag peptides, the percentage of CFSE^dim^ PD-1^+^ CD4 T cells was significantly higher than PD-1^−^ CD4 T cells (PD-1^+^ vs PD-1^−^: 1.48±0.32 vs 0.29±0.06) (Figure S3B). The percentage of CFSE^dim^ PD-1^+^ CD8 T cells was also significantly higher than PD-1^−^ CD8 T cells (PD-1^+^ vs PD-1^−^: 2.45±1.32 vs 0.49±0.138, Figure S3B). Furthermore, CFSE^dim^ CD4 and CD8 T cells predominantly expressed PD-1 (CD4 vs CD8: 82.3±11.2 vs 85.5±6.3) (Figure S3C). Thus, both CD4^+^ and CD8^+^ T cells that are either undergoing proliferation or those that have previously undergone proliferation preferentially express PD-1 during chronic infection.

### Relationship between proliferating PD-1^+^T cells and plasma viremia

**Figure 4:**
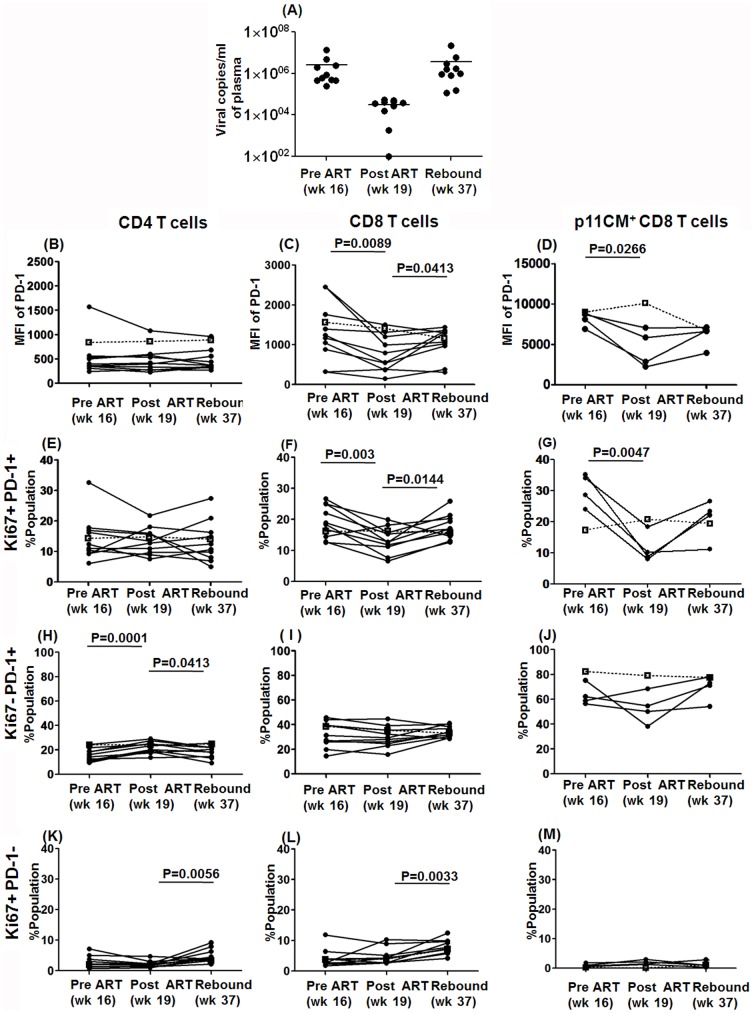
Alteration in the frequencies of circulating Ki67^+^PD-1^+^, Ki67^−^PD-1^+^, Ki67^+^PD-1^−^ T cell subsets in ART treated animals during chronic SIVmac239 infection. Ten rhesus macaques received ART for 28 days initiated at 112 days post infection. (A) level of plasma viral load prior to (wk 16), during (wk 19) and after cessation of ART treatment (wk37) at chronic SIV infection. The MFI of PD-1, the frequencies of Ki67^+^PD-1^+^, Ki67^−^PD-1^+^, and Ki67^+^PD-1^−^ on CD4 (B, E, H and K), CD8 (C, F, I, and L), and p11CM^+^ CD8^+^ T cells (D, G, J, and M) before and after the ART treatment at chosen time points. The p-values shown are the result of paired t-test analysis. For purpose of control, values from the 10 non-ART treated control monkeys (CD4 and CD8 T cells) or the 3 non-ART treated controls (p11CM^+^CD8 T cells) were averaged and shown as a discontinuous line in panels B-M.

In efforts to determine whether the increased frequencies of proliferating PD-1^+^ CD4 and CD8 T cells were secondary to the presence of relatively high levels of persisting viremia, we examined the frequencies of PD-1 expression by Ki67^+^ PD-1^+^ CD4^+^ and CD8^+^ T cells in the blood from a subset of 10 animals before, during and after a 28 day (4 wk) course of ART treatment during the chronic phase of SIV infection. The initiation of antiretroviral therapy (at 16 wk) as expected, resulted in a rapid reduction of viral load (as seen at 19 wk) and following cessation of ART showed a rebound to pre-ART levels at 37 wk (Figure 4A). We found a significant decrease in the level of PD-1 density and the frequencies of Ki67^+^PD-1**^+^** expression by both total and SIV-specific CD8 T cells, in response to the decline in viral load following initiation of ART (Figure 4C, D, F and G). Although these levels were not fully restored to pre ART levels after viral rebound, they essentially increased again to significant levels. For purposes of control the mean values of the non-treated group are indicated as a discontinuous line in Figures 4B-M. Of interest was the finding that the frequencies of Ki67 negative PD-1**^+^** CD8 T cells did not vary during the treatment except for the variations noted for the p11CM^+^ antigen specific CD8^+^ T cells (Figure 4 I and J), while the frequencies of Ki67^+^PD-1^−^ CD8 T cells showed no decrease upon ART initiation but a significant increase upon viral rebound (Figure 4L).

In contrast, the frequency of Ki67^+^PD-1**^+^** CD4 T cells did not change significantly (Figure 4B and E) over the course of ART. There was a slight increase in the Ki67 negative PD-1**^+^** CD4 T cells during ART and an increase in Ki67^+^PD-1 negative CD4 T cells correlating with the viral rebound, similar to CD8 T cells. These findings demonstrate that the proliferative status of PD-1^+^ CD8 T cell subsets was preferentially affected by viral antigen during the chronic phase.

### PD-1 expression by different subsets of CD4 and CD8 T cells in blood

**Figure 5:**
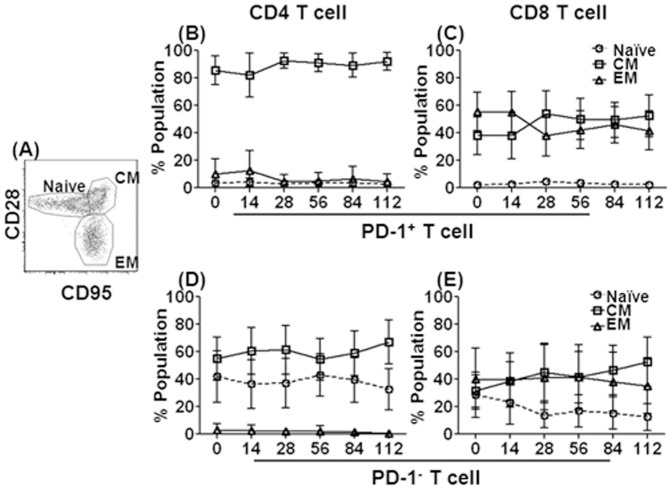
Longitudinal analysis of circulating PD-1 positive and negative naïve, CM and EM CD4 and CD8 T cells. The gating strategy and representative flow cytometry profile of naïve (CD28^int^ CD95^−^), central memory (CD28^+^ CD95^+^) and effector memory (CD28^−^ CD95^+^) T cells (A). The frequencies of naïve and memory subsets are shown in PD-1**^+^** CD4 (B), PD-1**^+^** CD8 (C), PD-1^−^ CD4(D) and PD-1^−^ CD8 (E) T cells prior to and following SIV infection. The data represents mean values and standard deviations from whole blood samples from 20 animals for each time point.

To define the frequencies of PD-1 expressing subsets of CD4 and CD8 T cells, we analyzed naïve (CD28^int^ CD95^−^), central memory (CD28^+^ CD95^+^) and effector memory (CD28^−^ CD95^+^) T cells [22](Figure 5A). The vast majority of PD-1^+^ CD4 and CD8 T cells were of the memory phenotype (94.6%±0.9 and 91.9%±1.3, respectively) prior to as well as after infection (Figure 5B and C). PD-1^+^ CD4 T cells represented essentially central memory T cells, while PD-1^+^ CD8 T cells were a mix of central and effector memory T cells with only minor variations over time post infection. In contrast, PD-1 negative CD4 T cells comprised naïve and central memory T cells, reflecting low levels of circulating effector memory cells (Figure 5D). The PD-1 negative CD8 T cells represented a mix of naïve and memory subsets (Figure 5E), with a progressive decrease of naïve CD8 T cells. Of interest though, was the fact that PD-1 expression on these T cells is unlikely to be specifically related to persistent antigenic stimulation during chronic infection, since only a small fraction of these cells is specific for SIV antigens. It is also possible that PD-1^+^ CD8 T cells have a higher immediate effector potential than CD4 T cells and might circulate through the extra-lymphoid effector sites of the body [30] in both healthy and SIV infected animals.

**Figure 6:**
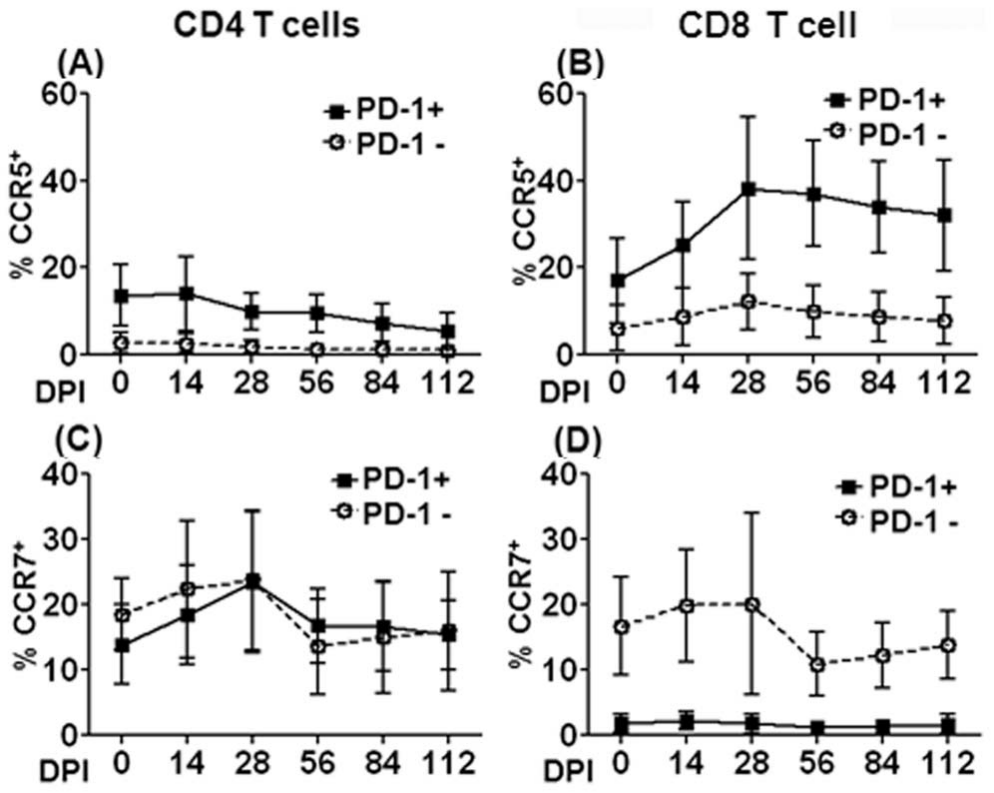
Longitudinal analysis of homing markers on PD-1 positive and negative CD4 and CD8 T cells in blood samples from SIV-infected macaques. Percentage of CCR5^+^ (A and B) and CCR7+ (C and D) cells in the blood of SIV infected rhesus macaques that did and did not express PD-1 were monitored by standard flow cytometry. Data are shown as mean ± SD. The data represents mean values and standard deviations from whole blood samples from 20 animals for each time point.

For additional definition of these T cell subsets, we analyzed the expression of CCR5 and CCR7 relevant to tissue trafficking on PD-1^+^ and PD-1^−^ T cells in whole blood. The tissue-homing molecules CCR5 involved in migration to extra-lymphoid effector sites and activation [31] was expressed at higher levels on CD4 and CD8 T cells that were positive for PD-1, compared to those that were negative (Figure 6, A and B). In CD4 T cells though, the difference in CCR5 expression between PD-1^+^ and PD-1 negative cells gradually decreased from pre infection to 4 months post infection, suggesting elimination of these cells or increased extravasation of this subset into tissues. In CD8 T cells, the frequency of CCR5 expression by PD-1^+^ cells peaked at 28 day post infection followed by a slow decline, but levels were markedly higher than pre-infection levels. This increased expression of CCR5 represents an increase in the level of CD8^+^ T cell activation during chronic infection but may also suggest a diminished capacity of CCR5^+^ CD8 T cells to traffic from the blood into tissues in response to chemokines such as RANTES and MIP-1α [32]. CCR7, a homing marker for lymphoid tissue is primarily expressed on naïve and central memory T cells, while its expression diminishes during effector differentiation [33]. Both PD-1^+^ and PD-1^−^ CD4 T cells expressed similar levels of CCR7 at all times pre and post-SIV infection (Figure 6C), with a transient increase of their frequencies by day 28 p.i. In contrast, CCR7 expression was undetectable on PD-1^+^ as compared with PD-1 negative CD8^+^ T cells (Figure 6D), suggesting that PD-1 expressing CD8 T cells may be highly differentiated and may have the ability to move into peripheral sites in response to inflammatory chemokines in both physiological and pathological environment.

## Discussion

A high density of PD-1 expression by virus-specific CD8 T cells has been reported in several chronic viral infections and linked to T cell exhaustion, characterized by poor proliferative capacity and the loss of ability to produce cytokines, during persistent viral infection. The in vivo immunological “rescue” effect of PD-1 blockade utilizing the administration of a partially humanized anti-PD1 monoclonal antibody (clone EH12-1540) was shown to lead to enhanced viral control both in mice [3], and the nonhuman primate models of human AIDS [34], [35]. These findings underscore the importance of PD-1 mediated effector inhibition in vivo, designed to limit T cell activation and the ensuing inflammatory consequences. This mechanism is not limited to chronic viral infection but likely results from exposure to chronic high antigenemia, also seen in cancer, leading to ineffective host responses in spite of tumors expressing readily detectable immunogens in the host. Recent reports from clinical trials have shown that PD-1 blockade in patients with terminal cancers comprising colon carcinoma and melanoma achieved clinical benefit as well as remission in a small percentage of patients [7], [8].

However, recent reports have suggested that PD-1 expression may also serve as a predictive marker for immune activation and potential disease progression [26] which led us to examine our cohort of SIV infected macaques in more detail to investigate such a hypothesis. Similar to previous studies, we found that SIV-specific CD8 T cells showed poor proliferative activity during chronic SIV infection, which correlated with increased PD-1 expression on select SIV specific T cells. However, PD-1 upregulation was observed on far more cells than the number of commonly detected SIV specific T cells in vivo, leading to the question of the relevance of PD-1 expression on bystander CD4 and CD8 T cells. The co-ordinate analysis of PD-1 expression with Ki67, a marker of cell proliferation, led to a series of observations. First, while the frequency of non-proliferating total PD-1 expressing T cells did not increase during chronic infection, the proportion of PD-1^+^ proliferating T cells increased. Second, our correlative analysis suggests that sustained PD-1 expression directly correlates with the proliferation status of total CD4 and CD8 T cells in animals with high sustained viral loads, suggesting that PD-1 likely represents an additional activation marker induced during both SIV [36] and HIV infection [12], [36], [37]. What remains to be elucidated in these bystander PD1^+^ T cells is the exact role of PD-1 and whether PD-1 on these cells is being engaged by its ligands or not. Based on the results of the p11CM tetramer positive cells, we submit that for CD8 T cells at least, the majority of the cells in which PD-1 has been upregulated are not antigen specific. Next, the mechanisms by which PD-1 is being upregulated in bystander T cells during SIV infection may have less to do with TCR engagement but more likely represent increased homeostatic proliferation due to higher levels of immune activation and cytokine release [29]. It would appear that on these cells, PD-1 expression does not necessarily herald immune exhaustion but instead adds to the list of cell surface markers associated with activation. Nonetheless, whether such up-regulation leads to interaction with PD-1 ligands and T cell dysfunction remains an important mechanism to investigate. Of note, in healthy individuals, most PD-1 expressing cells show effector memory phenotype rather than exhausted phenotype in CD8^+^ T cells [11]. In fact, PD-1 expression also depends on the differentiation stage of T cells [12]. Our data also showed that there was no significant augmentation of PD-1 expression on CD4^+^ and CD8^+^ T cells during any time point post infection, consistent with our previous report [22]. One may speculate that the up-regulation of PD-1 on activated CD4^+^ and CD8^+^ T cells may be part of the homeostasis of differentiated or activated T cells to render them prone to apoptosis, leading to limit growth and reactivity in both healthy and disease condition [11].

One interesting finding of our in vivo study is that circulating PD-1^+^ CD8^+^ T cells as compared with CD4^+^ T cells are more sensitive to antigen-induced proliferation. First, the frequency of proliferating PD-1^+^CD8^+^ T cells peak during the acute phase of infection followed by a decline at the initiation of the chronic phase paralleling plasma viral loads, while PD-1^+^CD4^+^ T cells gradually increase during the course of infection. Second, alteration in the levels of plasma viral antigen during ART affects PD-1 expression and the frequencies of proliferating PD-1^+^ CD8^+^ T cells, but not CD4^+^ T cells. Although there is currently no direct evidence to substantiate this hypothesis, it is reasoned that activated PD-1^+^ CD4^+^ T cells might be more affected by immune mediators during chronic infection. For example, PD-1 expression was also induced by common γ-chain-associated cytokines, such as IL-2, IL-7, IL-15 and IL-21 each of which is associated with T cell activation [9]. Type I IFNs including IFN-α, which limits viral replication, also enhanced PD-1 expression [38]. Moreover, in light of our recent findings on the accumulation of specialized PD-1^hi^ CD4 T cells in lymphoid tissues during SIV infection [17], further investigations are warranted.

Another interesting finding of our study is that circulating PD-1^+^ T cells have disparate expression of tissue-trafficking markers. Our data showed that circulating PD-1^+^ CD4 and CD8 T cells may have the potential ability to traffic into inflammatory sites as activated cells. Particularly, PD-1^+^ CD8 T cells express a CCR5^+^ CCR7^−^ phenotype, relative to the PD-1 negative counterpart. On the other hand, the decline in CCR5^+^ PD-1^+^ CD4 T cells populations during chronic infection may reflect the fact that they can be major targets for HIV/SIV replication during primary infection [39]–[41].

In summary, SIV-specific CD8 T cells showed a negative correlation between the densities of PD-1 and Ki67 expression throughout the chronic phase of infection. However, proliferating and proliferated total CD4 and CD8 T cells preferentially expressed PD-1 on their surface in our study. Viral suppression and rebound after ART has been shown to parallel changes in the proportion of proliferating PD-1^+^ CD8^+^ T cell populations, but not CD4 T cells. Interestingly, circulating PD-1^+^ T cells showed disparate expression of tissue-trafficking molecules, compared with PD-1^−^ counterparts. These findings imply that PD-1 levels on CD4 and CD8 T cells, excluding virus-specific CD8 T cells, does not by itself serve as a marker for a state of cellular exhaustion during chronic SIV infection.

## Supporting Information

Figure S1 **Longitudinal analysis of plasma viremia and PD-1 expression on CD4, CD8, CD3^+^ NKG2a^+^ and CD3^−^ NKG2a^+^ cells in blood and tissues of twenty SIVmac239-infected rhesus macaques.** Plasma viral load (A) and MFI (B to Q) of PD-1 expression were monitored by flow cytometric analysis of CD4 (B to E), CD8 (F to I), CD3^+^ NKG2a^+^ (J to M), and CD3^−^ NKG2a^+^ (N to Q) cells in blood, bone marrow, lymph node and colorectal tissues. Data are shown as mean ± SD. Whole blood, bone marrow, lymph node, and colorectal cell samples from twenty animals were used for the analyses, except for the lymph node at 0 dpi (n = 13).
(TIF)

Figure S2 **Longitudinal analysis of PD-1 and Ki67 expression on CD3^+^ NKG2a^+^ and CD3^−^ NKG2a^+^ cells in blood and tissues of twenty SIVmac239-infected rhesus macaques.** The frequency of PD-1^+^ Ki67^+^, PD-1^+^ Ki67^−^ and PD-1^−^ Ki67^+^ cells prior to and following SIVmac239 infection in whole blood, bone marrow, lymph node, and colorectal tissues by CD3^+^ NKG2a^+^ (A, C, E and G) and CD3^−^ NKG2a^+^ (B, D, F, and H) cells. Whole blood, bone marrow, lymph node, and colorectal cell samples from twenty animals were used for the analyses, except for the lymph node at 0 dpi (n = 13).
(TIF)

Figure S3 **PD-1 expressing CD4 and CD8 T cells show proliferation status (CFSE^dim^ cells), compared to PD-1^−^ cells.** Proliferation of live-gated PD-1^+ or −^ T cells after a 6 day in vitro stimulation was assessed by flowcytometry (A). PBMCs labeled with CFSE were re-stimulated with either ovalbumin (control) or a pool of overlapping SIVgag peptides (1 µg/ml) (B). Each dot represents a response of a CD4 and CD8 T cell from PBMCs of seventeen rhesus macaques chronically infected with SIVmac239. Percentage of PD-1 expression on CFSE^dim^ CD4 and CD8 T cells (C).
(TIF)

## References

[1] Keir ME, Butte MJ, Freeman GJ, Sharpe AH (2008) PD-1 and its ligands in tolerance and immunity. Annual Review of Immunology 26: 677–704 10.1146/annurev.immunol.26.021607.090331. 10.1146/annurev.immunol.26.021607.090331 PMC10637733 18173375

[2] Agata Y, Kawasaki A, Nishimura H, Ishida Y, Tsubata T, et al (1996) Expression of the PD-1 antigen on the surface of stimulated mouse T and B lymphocytes. International Immunology 8: 765–772. 8671665 10.1093/intimm/8.5.765

[3] Barber DL, Wherry EJ, Masopust D, Zhu B, Allison JP, et al (2006) Restoring function in exhausted CD8 T cells during chronic viral infection. Nature 439: 682–687 10.1038/nature04444. 16382236 10.1038/nature04444

[4] Day CL, Kaufmann DE, Kiepiela P, Brown JA, Moodley ES, et al (2006) PD-1 expression on HIV-specific T cells is associated with T-cell exhaustion and disease progression. Nature 443: 350–354 10.1038/nature05115. 16921384 10.1038/nature05115

[5] Golden-Mason L, Palmer B, Klarquist J, Mengshol JA, Castelblanco N, et al (2007) Upregulation of PD-1 expression on circulating and intrahepatic hepatitis C virus-specific CD8+ T cells associated with reversible immune dysfunction. Journal of Virology 81: 9249–9258 10.1128/JVI.00409-07. 17567698 10.1128/JVI.00409-07 PMC1951397

[6] Yao ZQ, King E, Prayther D, Yin D, Moorman J (2007) T cell dysfunction by hepatitis C virus core protein involves PD-1/PDL-1 signaling. Viral Immunology 20: 276–287 10.1089/vim.2006.0096. 17603844 10.1089/vim.2006.0096

[7] Topalian SL, Hodi FS, Brahmer JR, Gettinger SN, Smith DC, et al (2012) Safety, activity, and immune correlates of anti-PD-1 antibody in cancer. The New England Journal of Medicine 366: 2443–2454 10.1056/NEJMoa1200690. 22658127 10.1056/NEJMoa1200690 PMC3544539

[8] Brahmer JR, Tykodi SS, Chow LQ, Hwu WJ, Topalian SL, et al (2012) Safety and activity of anti-PD-L1 antibody in patients with advanced cancer. The New England Journal of Medicine 366: 2455–2465 10.1056/NEJMoa1200694. 22658128 10.1056/NEJMoa1200694 PMC3563263

[9] Kinter AL, Godbout EJ, McNally JP, Sereti I, Roby GA, et al (2008) The common gamma-chain cytokines IL-2, IL-7, IL-15, and IL-21 induce the expression of programmed death-1 and its ligands. . Journal of Immunology (Baltimore, Md.: 1950) 181: 6738–6746. 10.4049/jimmunol.181.10.6738 18981091

[10] Salisch NC, Kaufmann DE, Awad AS, Reeves RK, Tighe DP, et al (2010) Inhibitory TCR coreceptor PD-1 is a sensitive indicator of low-level replication of SIV and HIV-1. Journal of Immunology (Baltimore, Md.: 1950) 184: 476–487 10.4049/jimmunol.0902781; 10.4049/jimmunol.0902781. 10.4049/jimmunol.0902781 PMC2810496 19949078

[11] Duraiswamy J, Ibegbu CC, Masopust D, Miller JD, Araki K, et al (2011) Phenotype, function, and gene expression profiles of programmed death-1(hi) CD8 T cells in healthy human adults. Journal of Immunology (Baltimore, Md.: 1950) 186: 4200–4212 10.4049/jimmunol.1001783. 10.4049/jimmunol.1001783 PMC3723805 21383243

[12] Sauce D, Almeida JR, Larsen M, Haro L, Autran B, et al (2007) PD-1 expression on human CD8 T cells depends on both state of differentiation and activation status. AIDS (London, England) 21: 2005–2013 10.1097/QAD.0b013e3282eee548. 10.1097/QAD.0b013e3282eee548 17885290

[13] Vollbrecht T, Brackmann H, Henrich N, Roeling J, Seybold U, et al (2010) Impact of changes in antigen level on CD38/PD-1 co-expression on HIV-specific CD8 T cells in chronic, untreated HIV-1 infection. Journal of Medical Virology 82: 358–370 10.1002/jmv.21723. 20087935 10.1002/jmv.21723

[14] Ssewanyana I, Baker CA, Ruel T, Bousheri S, Kamya M, et al (2009) The distribution and immune profile of T cell subsets in HIV-infected children from uganda. AIDS Research and Human Retroviruses 25: 65–71 10.1089/aid.2008.0138. 19182922 10.1089/aid.2008.0138 PMC2858297

[15] Rueda CM, Velilla PA, Chougnet CA, Montoya CJ, Rugeles MT (2012) HIV-induced T-cell activation/exhaustion in rectal mucosa is controlled only partially by antiretroviral treatment. PloS One 7: e30307 10.1371/journal.pone.0030307. 22276176 10.1371/journal.pone.0030307 PMC3261885

[16] Nakanjako D, Ssewanyana I, Mayanja-Kizza H, Kiragga A, Colebunders R, et al (2011) High T-cell immune activation and immune exhaustion among individuals with suboptimal CD4 recovery after 4 years of antiretroviral therapy in an african cohort. BMC Infectious Diseases 11: 43 10.1186/1471-2334-11-43. 21299909 10.1186/1471-2334-11-43 PMC3065409

[17] Hong JJ, Amancha PK, Rogers K, Ansari AA, Villinger F (2012) Spatial alterations between CD4(+) T follicular helper, B, and CD8(+) T cells during simian immunodeficiency virus infection: T/B cell homeostasis, activation, and potential mechanism for viral escape. Journal of Immunology (Baltimore, Md.: 1950) 188: 3247–3256 10.4049/jimmunol.1103138. 10.4049/jimmunol.1103138 PMC3311732 22387550

[18] Kaizu M, Borchardt GJ, Glidden CE, Fisk DL, Loffredo JT, et al (2007) Molecular typing of major histocompatibility complex class I alleles in the indian rhesus macaque which restrict SIV CD8+ T cell epitopes. Immunogenetics 59: 693–703 10.1007/s00251-007-0233-7. 17641886 10.1007/s00251-007-0233-7

[19] Loffredo JT, Sidney J, Bean AT, Beal DR, Bardet W, et al (2009) Two MHC class I molecules associated with elite control of immunodeficiency virus replication, mamu-B*08 and HLA-B*2705, bind peptides with sequence similarity. . Journal of Immunology (Baltimore, Md.: 1950) 182: 7763–7775 10.4049/jimmunol.0900111. 10.4049/jimmunol.0900111 PMC2701622 19494300

[20] Amara RR, Villinger F, Altman JD, Lydy SL, O′Neil SP, et al (2001) Control of a mucosal challenge and prevention of AIDS by a multiprotein DNA/MVA vaccine. Science New York, N.Y. 292: 69–74. 11393868 10.1126/science.1058915

[21] Veazey RS, Rosenzweig M, Shvetz DE, Pauley DR, DeMaria M, et al (1997) Characterization of gut-associated lymphoid tissue (GALT) of normal rhesus macaques. Clinical Immunology and Immunopathology 82: 230–242. 9073546 10.1006/clin.1996.4318

[22] Onlamoon N, Rogers K, Mayne AE, Pattanapanyasat K, Mori K, et al (2008) Soluble PD-1 rescues the proliferative response of simian immunodeficiency virus-specific CD4 and CD8 T cells during chronic infection. Immunology 124: 277–293. 18266718 10.1111/j.1365-2567.2007.02766.x PMC2566632

[23] Sumpter B, Dunham R, Gordon S, Engram J, Hennessy M, et al (2007) Correlates of preserved CD4(+) T cell homeostasis during natural, nonpathogenic simian immunodeficiency virus infection of sooty mangabeys: Implications for AIDS pathogenesis. . Journal of Immunology (Baltimore, Md.: 1950) 178: 1680–1691. 10.4049/jimmunol.178.3.1680 17237418

[24] Gordon SN, Klatt NR, Bosinger SE, Brenchley JM, Milush JM, et al (2007) Severe depletion of mucosal CD4+ T cells in AIDS-free simian immunodeficiency virus-infected sooty mangabeys. . Journal of Immunology (Baltimore, Md.: 1950) 179: 3026–3034. 10.4049/jimmunol.179.5.3026 PMC2365740 17709517

[25] Berhanu D, Mortari F, De Rosa SC, Roederer M (2003) Optimized lymphocyte isolation methods for analysis of chemokine receptor expression. Journal of Immunological Methods 279: 199–207. 12969561 10.1016/s0022-1759(03)00186-8

[26] Wherry EJ (2011) T cell exhaustion. Nature Immunology 12: 492–499. 21739672 10.1038/ni.2035

[27] Soares A, Govender L, Hughes J, Mavakla W, de Kock M, et al (2010) Novel application of Ki67 to quantify antigen-specific in vitro lymphoproliferation. Journal of Immunological Methods 362: 43–50 10.1016/j.jim.2010.08.007. 20800066 10.1016/j.jim.2010.08.007 PMC2989440

[28] Shedlock DJ, Talbott KT, Morrow MP, Ferraro B, Hokey DA, et al (2010) Ki-67 staining for determination of rhesus macaque T cell proliferative responses ex vivo. Cytometry.Part A: The Journal of the International Society for Analytical Cytology 77: 275–284 10.1002/cyto.a.20857. 20104580 10.1002/cyto.a.20857 PMC2939446

[29] Petrovas C, Price DA, Mattapallil J, Ambrozak DR, Geldmacher C, et al (2007) SIV-specific CD8+ T cells express high levels of PD1 and cytokines but have impaired proliferative capacity in acute and chronic SIVmac251 infection. Blood 110: 928–936. 17440051 10.1182/blood-2007-01-069112 PMC1924769

[30] Pitcher CJ, Hagen SI, Walker JM, Lum R, Mitchell BL, et al (2002) Development and homeostasis of T cell memory in rhesus macaque. . Journal of Immunology (Baltimore, Md.: 1950) 168: 29–43. 10.4049/jimmunol.168.1.29 11751943

[31] Wong MM, Fish EN (2003) Chemokines: Attractive mediators of the immune response. Seminars in Immunology 15: 5–14. 12495636 10.1016/s1044-5323(02)00123-9

[32] Jones KL, Maguire JJ, Davenport AP (2011) Chemokine receptor CCR5: From AIDS to atherosclerosis. . British Journal of Pharmacology 162 1453–1469 10.1111/j.1476-5381.2010.01147.x;10.1111/j.1476-5381.2010.01147.x. 21133894 10.1111/j.1476-5381.2010.01147.x PMC3057285

[33] Forster R, Davalos-Misslitz AC, Rot A (2008) CCR7 and its ligands: Balancing immunity and tolerance. Nature Reviews.Immunology 8: 362–371 10.1038/nri2297. 10.1038/nri2297 18379575

[34] Dyavar Shetty R, Velu V, Titanji K, Bosinger SE, Freeman GJ, et al. (2012) PD-1 blockade during chronic SIV infection reduces hyperimmune activation and microbial translocation in rhesus macaques. The Journal of Clinical Investigation 122: : 1712-1716.10.1172/JCI60612; 10.1172/JCI60612. 10.1172/JCI60612 PMC3336983 22523065

[35] Velu V, Titanji K, Zhu B, Husain S, Pladevega A, et al (2009) Enhancing SIV-specific immunity in vivo by PD-1 blockade. Nature 458: 206–210 10.1038/nature07662. 19078956 10.1038/nature07662 PMC2753387

[36] Hokey DA, Johnson FB, Smith J, Weber JL, Yan J, et al (2008) Activation drives PD-1 expression during vaccine-specific proliferation and following lentiviral infection in macaques. European Journal of Immunology 38: 1435–1445. 18389475 10.1002/eji.200737857 PMC2996615

[37] Tendeiro R, Foxall RB, Baptista AP, Pinto F, Soares RS, et al.. (2012) PD-1 and its ligand PD-L1 are progressively up-regulated on CD4 and CD8 T-cells in HIV-2 infection irrespective of the presence of viremia. AIDS (London,England). 10.1097/QAD.0b013e32835374db. 10.1097/QAD.0b013e32835374db 22441249

[38] Terawaki S, Chikuma S, Shibayama S, Hayashi T, Yoshida T, et al (2011) IFN-alpha directly promotes programmed cell death-1 transcription and limits the duration of T cell-mediated immunity. . Journal of Immunology (Baltimore, Md.: 1950) 186: 2772–2779 10.4049/jimmunol.1003208. 10.4049/jimmunol.1003208 21263073

[39] Centlivre M, Sala M, Wain-Hobson S, Berkhout B (2007) In HIV-1 pathogenesis the die is cast during primary infection. AIDS London, England 21: 1–11 10.1097/QAD.0b013e3280117f7f. 17148962 10.1097/QAD.0b013e3280117f7f

[40] Doms RW (2001) Chemokine receptors and HIV entry. AIDS London, England 15 Suppl 1S34–5. 11403007 10.1097/00002030-200102001-00051

[41] Sina ST, Ren W, Cheng-Mayer C (2011) Coreceptor use in nonhuman primate models of HIV infection. Journal of Translational Medicine 9 Suppl 1S7 10.1186/1479-5876-9-S1-S7. 21284906 10.1186/1479-5876-9-S1-S7 PMC3105507

