# Supplementary figures and images for: Re-Evaluation of PD-1 Expression by T Cells as a Marker for Immune Exhaustion during SIV Infection
Source: PLoS One. 2013 Mar 28;8(3):e60186. doi: 10.1371/journal.pone.0060186 (PMC3610666; doi:10.1371/journal.pone.0060186)

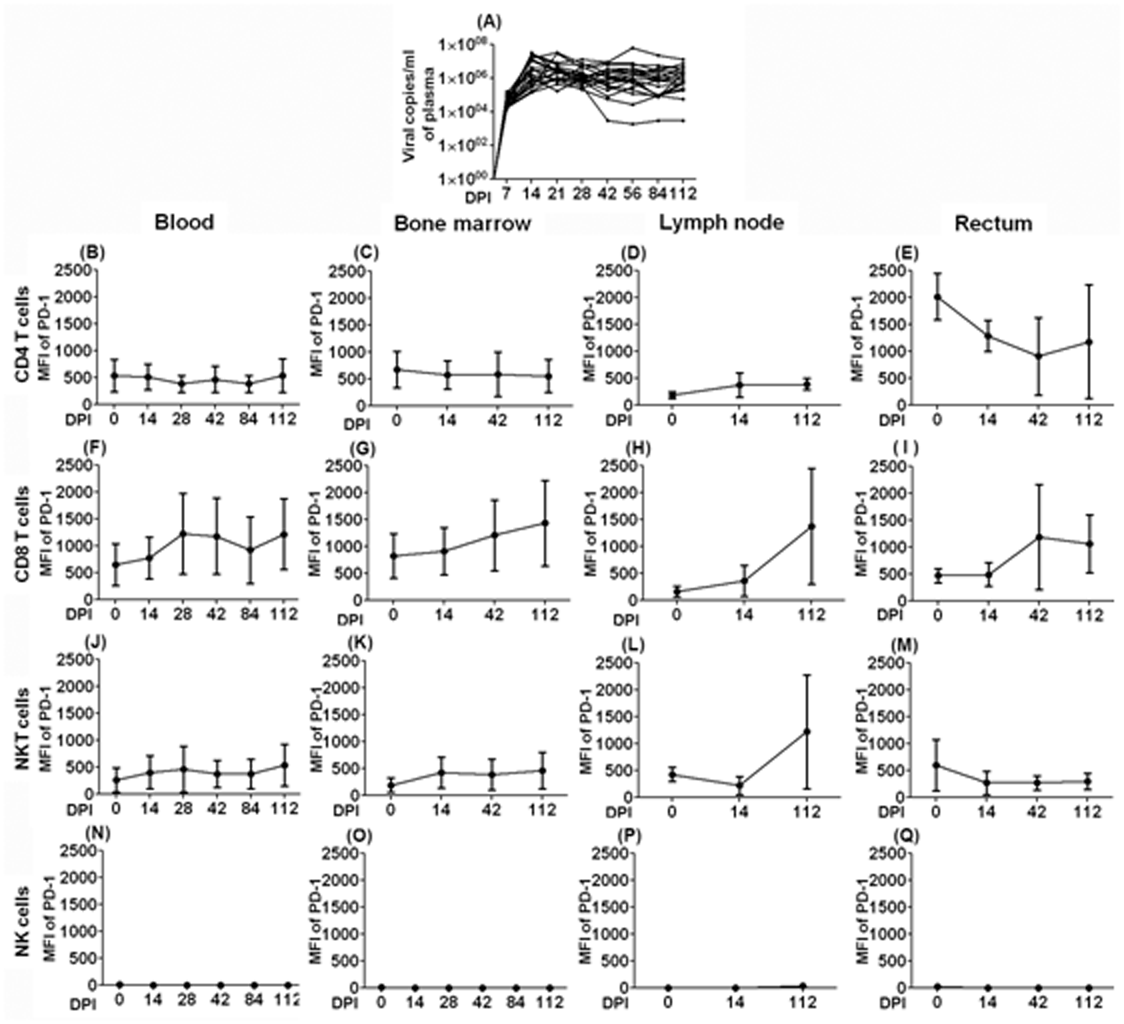

Supplement: Figure S1 — Longitudinal analysis of plasma viremia and PD-1 expression on CD4, CD8, CD3+ NKG2a+ and CD3− NKG2a+ cells in blood and tissues of twenty SIVmac239-infected rhesus macaques. Plasma viral load (A) and MFI (B to Q) of PD-1 expression were monitored by flow cytometric analysis of CD4 (B to E), CD8 (F to I), CD3+ NKG2a+ (J to M), and CD3− NKG2a+ (N to Q) cells in blood, bone marrow, lymph node and colorectal tissues. Data are shown as mean ± SD. Whole blood, bone marrow, lymph node, and colorectal cell samples from twenty animals were used for the analyses, except for the lymph node at 0 dpi (n = 13). (TIF) [file pone.0060186.s001.tif]

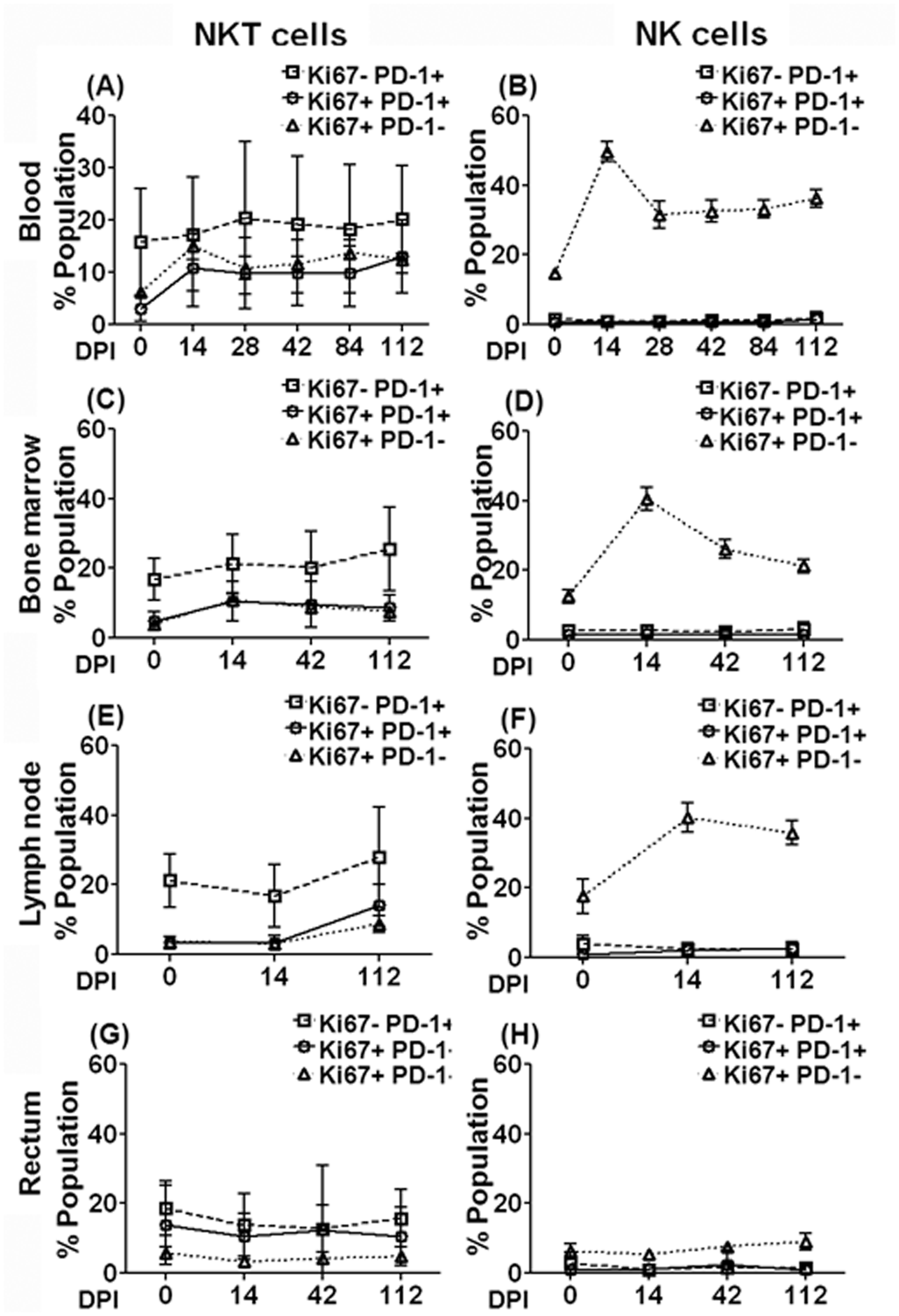

Supplement: Figure S2 — Longitudinal analysis of PD-1 and Ki67 expression on CD3+ NKG2a+ and CD3− NKG2a+ cells in blood and tissues of twenty SIVmac239-infected rhesus macaques. The frequency of PD-1+ Ki67+, PD-1+ Ki67− and PD-1− Ki67+ cells prior to and following SIVmac239 infection in whole blood, bone marrow, lymph node, and colorectal tissues by CD3+ NKG2a+ (A, C, E and G) and CD3− NKG2a+ (B, D, F, and H) cells. Whole blood, bone marrow, lymph node, and colorectal cell samples from twenty animals were used for the analyses, except for the lymph node at 0 dpi (n = 13). (TIF) [file pone.0060186.s002.tif]

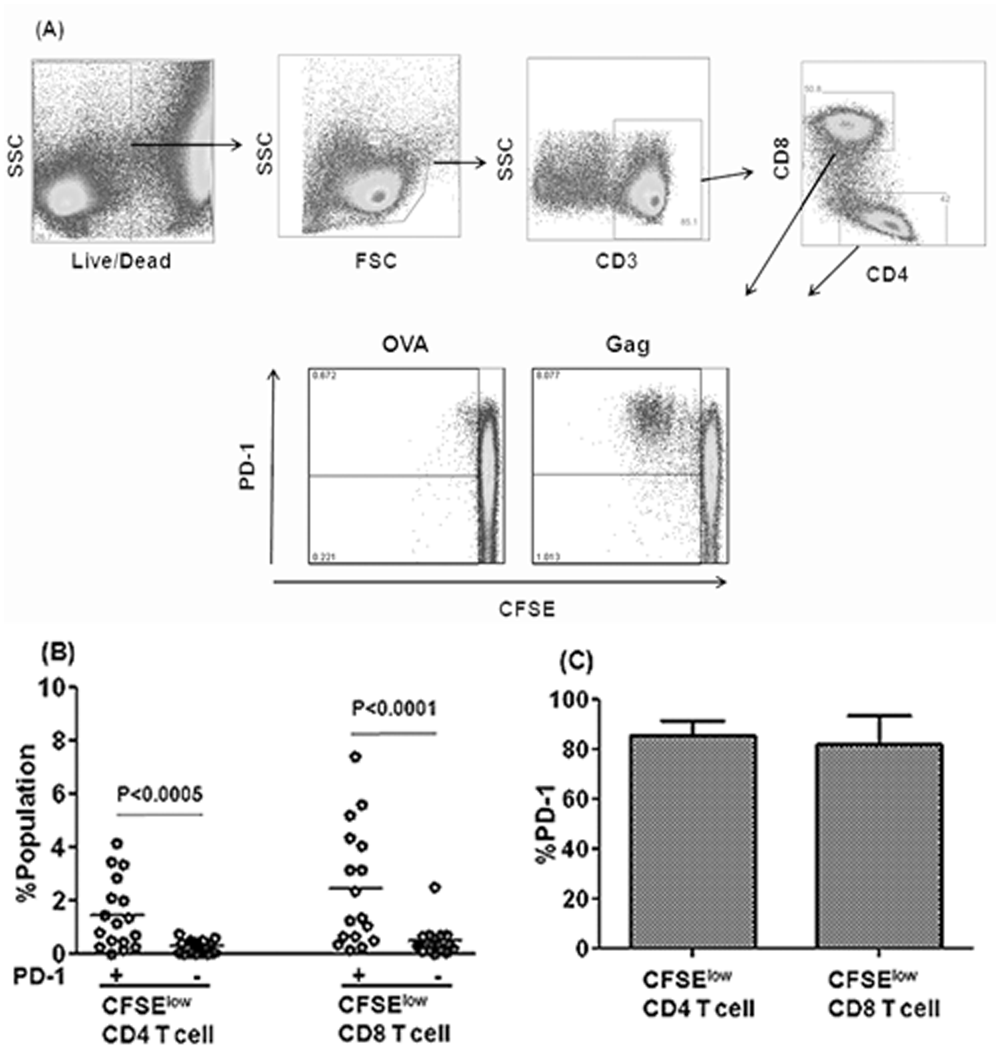

Supplement: Figure S3 — PD-1 expressing CD4 and CD8 T cells show proliferation status (CFSEdim cells), compared to PD-1− cells. Proliferation of live-gated PD-1+ or − T cells after a 6 day in vitro stimulation was assessed by flowcytometry (A). PBMCs labeled with CFSE were re-stimulated with either ovalbumin (control) or a pool of overlapping SIVgag peptides (1 µg/ml) (B). Each dot represents a response of a CD4 and CD8 T cell from PBMCs of seventeen rhesus macaques chronically infected with SIVmac239. Percentage of PD-1 expression on CFSEdim CD4 and CD8 T cells (C). (TIF) [file pone.0060186.s003.tif]
